# Neural computations underlying social risk sensitivity

**DOI:** 10.3389/fnhum.2012.00213

**Published:** 2012-08-02

**Authors:** Nina Lauharatanahirun, George I. Christopoulos, Brooks King-Casas

**Affiliations:** ^1^Virginia Tech Carilion Research Institute, RoanokeVA, USA; ^2^Department of Psychology, Virginia Tech, BlacksburgVA, USA; ^3^Research Service Line, Salem Veterans Affairs Medical Center, SalemVA, USA; ^4^Nanyang Business School, Nanyang Technological UniversitySingapore; ^5^Culture Science Institute, Nanyang Technological UniversitySingapore; ^6^Department of Psychiatry, Virginia Tech Carilion School of Medicine, RoanokeVA, USA; ^7^Virginia Tech - Wake Forest University School of Biomedical Engineering and Sciences, BlacksburgVA, USA

**Keywords:** fMRI, individual differences, risk, social neuroscience, trust

## Abstract

Under standard models of expected utility, preferences over stochastic events are assumed to be independent of the source of uncertainty. Thus, in decision-making, an agent should exhibit consistent preferences, regardless of whether the uncertainty derives from the unpredictability of a random process or the unpredictability of a social partner. However, when a social partner is the source of uncertainty, social preferences can influence decisions over and above pure risk attitudes (RA). Here, we compared risk-related hemodynamic activity and individual preferences for two sets of options that differ only in the social or non-social nature of the risk. Risk preferences in social and non-social contexts were systematically related to neural activity during decision and outcome phases of each choice. Individuals who were more risk averse in the social context exhibited decreased risk-related activity in the amygdala during non-social decisions, while individuals who were more risk averse in the non-social context exhibited the opposite pattern. Differential risk preferences were similarly associated with hemodynamic activity in ventral striatum at the outcome of these decisions. These findings suggest that social preferences, including aversion to betrayal or exploitation by social partners, may be associated with variability in the response of these subcortical regions to social risk.

## Introduction

A basic assumption of standard utility models (Von Neumann and Morgenstern, 1944) is that choices over uncertain outcomes are (or should be) completely uninfluenced by the source of the uncertainty. In other words, what matters is the distribution of previous outcomes and not the mechanism through which these outcomes were generated. For instance, faced with an investment option known to yield a 10% return, an agent should make the same investment decision regardless of whether the historical outcomes were determined by a die roll, a roulette wheel, a horse race, a market, or a human partner.

Trusting a social partner can be approached as a form of social investment involving risk. Certainly, trust implies investing a valued resource (be it money, time, emotions, or social capital) in another person or group, usually with the hope of reciprocation in the same or other form (Camerer and Weigelt, 1988). Thus, decisions to trust a social partner might be influenced by one's general attitude toward risk and be expected to scale with risk attitudes (RA) measured in non-social contexts. While a number of behavioral studies have provided empirical support for such a relationship (Karlan, 2005; Schechter, 2007), other work has suggested otherwise (Eckel and Wilson, 2004; Houser et al., 2010).

Trust may also be strongly influenced by additional parameters specific to social contexts. That is, individuals with similar non-social RA may still make different decisions within social trust exchanges (Eckel and Wilson, 2004; Karlan, 2005; Houser et al., 2010). Such parameters can function as trust-amplifiers or trust-inhibitors (Fehr, 2009). For instance, an agent might choose to invest in another out of pure altruism, even if the partner is entirely unknown (Charness and Rabin, 2002; Cox, 2004). Alternatively, social preferences may incorporate the disutility of interpersonal betrayal or exploitation, and thus inhibit trusting behavior, independent of risk, or regret aversion (Bohnet et al., 2008).

The tools of cognitive neuroscience have provided some evidence that trust and non-social risk preferences are neurobiologically dissociable. Intranasal administration of oxytocin increases trusting behavior while risk preferences remained unchanged (Kosfeld et al., 2005), suggesting that oxytocin is acting on parameters that are independent of risk. Neural correlates of risk and trust have previously been examined separately, identifying partially overlapping networks. Risk-related computations have been associated with activity in insular cortex (Preuschoff et al., 2008), amygdala (De Martino et al., 2006), striatum, anterior cingulate, and parietal cortex (Kuhnen and Knutson, 2005; Huettel et al., 2006; Christopoulos et al., 2009), while trust-related computations have been associated with activity in striatum, insula, and prefrontal cortex (McCabe et al., 2001; Delgado et al., 2005; King-Casas et al., 2005, 2008; Tomlin et al., 2006; Krueger et al., 2007; Chiu et al., 2008).

To examine the common and separable features of decision-making under risk in social and non-social contexts, we employed two investment tasks: one in which the outcome is determined by a social partner (trust game) and a second in which the outcome is determined by a random process (non-social gamble). The values and prior probabilities associated with different options were known to the participant and did not differ between the social and non-social conditions. We utilized a standard microeconomic behavioral model along with functional magnetic resonance (fMRI) to compare behavioral and neuronal differences between social and non-social conditions within subjects.

## Materials and methods

### Participants

Thirty-eight right-handed participants with a mean age of 26 yrs (SD = 7 yrs; *F* = 23) were recruited from the Houston metropolitan area. All participants consented to participation through a protocol approved by the Institutional Review Board of Baylor College of Medicine. Data from five participants were excluded prior to individual and group level analysis due to excessive head movement (>3 mm movement across the x, y, and z dimensions) (Friston et al., 1995), and three participants with extreme risk aversion parameters were excluded (detailed in Analysis section below).

### Experimental paradigm

**Figure 1:**
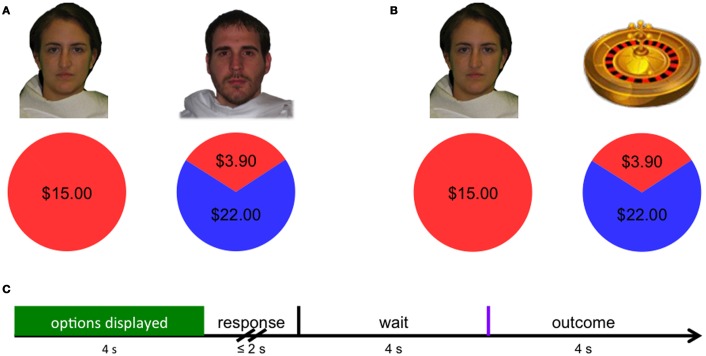
**(A)** In the social condition, subjects chose between keeping the original endowment or investing their endowment in a social partner. **(B)** In the non-social condition, subjects chose between keeping the original endowment or taking a gamble in which their payoff was determined by a non-social probabilistic mechanism (i.e., roulette wheel). **(C)** Decision phase activity was modeled across the first 4 s that options were presented (green), while outcome-related neural activity was modeled as the instantaneous response to the revealed outcome (purple).

Each participant made 86 decisions divided in two blocks, and order of blocks was balanced across subjects (see Figure 1). In a “social risk” block, individuals played 43 single-shot trust games (Camerer and Weigelt, 1988; Berg et al., 1995). All participants played the investor role. In each trial, participants were endowed with $5–$15, and could (i) keep the endowment (certain outcome) or (ii) invest the endowment in a second player (risky outcome). Trustees were depicted using neutral face images of actual trustees from a previous study who had consented for their images to be used as stimuli. Faces included both men and women from a variety of racial and ethnic backgrounds, and pairings of faces to options were randomized across trials to mitigate possible learning effects and bias. In the “non-social risk” block, individuals similarly received endowments between $5 and $15, and were able to either keep the endowment (certain outcome) or give up their endowment in order to accept a risky gamble (uncertain outcome). The outcome probabilities and values associated with risky outcomes in the social condition were determined based on behavior of a group of trustees making decisions in a previous session, and the distribution of outcomes in the “non-social” condition were matched to have the same mean (10.5), second moment (36.7), and third moment (−139.2). By explicitly revealing the probabilities associated with outcomes in both social and non-social conditions, this design removes a common confound of comparisons of risk and trust. That is, trust often involves outcomes for which probabilities are at least partly unknown, while decisions involving risk do not.

### Procedure

Participants were instructed that they would be making decisions to keep an endowment or invest their endowment in a risky option, either in another person (social risk condition) or in a gamble (non-social risk condition). In addition, participants were instructed that, in the social condition, a pie chart would indicate the average values and frequencies of actual repayments made by trustees in a previous session, and that repayments in the current session would be determined based on draws from this distribution. Participants were similarly instructed that in the non-social condition, a pie chart would indicate the values and probabilities of potential outcomes. Prior to scanning, participants were informed they would, in part, be compensated based on the outcomes of three randomly chosen trials.

### Analysis

RA expressed during social and non-social conditions were modeled using a constant relative risk aversion utility function (Pratt, 1964; Arrow, 1965; Holt and Laury, 2002), in which the utility of money *x*, for *x* > 0 is described by:

$$U(x)=\frac{x^{\left(1-r\right)}}{1-r}$$

where *x* represents the monetary value that the agent will receive and where *r* represents a risk attitude parameter such that *r* < 0 implies risk preference, *r* = 0 implies risk neutrality, and *r* > 0 implies risk aversion. When *r* = 1, we used *U*(*x*) = log(*x*) (Pratt, 1964; Arrow, 1965; Holt and Laury, 2002).

$$\mathrm{Pr}(choose\;A)=\frac{U_{A}^{1/\mu }}{U_{A}^{1/\mu }+U_{B}^{1/\mu }}$$

where μ varies between 0 and 1 and reflects the sensitivity of choices to the utilities associated with each option (Luce, 1959). The nlinfit function of Matlab (Mathworks, Natick, MA) was used to fit parameters of the model to actual choices. For each subject, the model was estimated 100 times for choices made in each condition (social and non-social). Three participants for whom estimates of *r* were outliers (mean greater than 1.5 standard deviations of the cohort) were excluded. These participants chose the risky option over 80% of the trials, while the remaining sample chose the risky option approximately half the time (*M*_non−social_ = 48.09%; SD_non−social_ = 21.64; *M*_social_ = 47.31%; SD_social_ = 0.19.24), placing these subjects over 1.5 standard deviations above the mean. Among the remaining 30 participants, the average *r* for each subject in each condition (social, non-social) were used as metrics of risk preference.

Functional images were acquired using a 3.0T Siemens Tim Trio with the following parameters: echo-planar imaging, gradient recalled echo; repetition time (TR) = 2 s; echo time (TE) = 30 ms; flip angle = 90°; 34 axial slices, 4.0 mm slice thickness, 220 × 220 mm field of view (FoV), 64 × 64 grid, resulting in voxels that were 3.4 × 3.4 × 4.0 mm, and hyperangulated slices were acquired at 30° from AC–PC. The structural scan was acquired using a high-resolution magnetization prepared rapid acquisition gradient echo sequence (TR = 1200 ms, TE = 2.66 ms, FoV = 245 mm, 1 mm slice thickness, 192 slices with spatial resolution of 1 × 1 × 1 mm^3^).

Images were preprocessed using SPM8 (Wellcome Department of Imaging Neuroscience, London, UK), using default values unless otherwise specified. Images were realigned, normalized using parameters derived from a segmented anatomical image coregistered to the mean EPI, and smoothed (6 × 6 × 6 mm). On the first level of the general linear model, three events of interest were modeled within each trial: 4 s presentation of options; onset of wait period; onset of decision outcome. Events were modeled separately for the following trial types and convolved with a canonical hemodynamic response function (HRF): risky social decision; certain social decision; risky non-social decision; certain non-social decision. Second level, random effects analyses were performed as specified below.

## Results

Estimates of RA in the social condition were strongly related to estimates of RA in the non-social condition across subjects (Spearman ϱ = 0.60, *p* < 0.001). In both conditions, participants as a whole were risk averse (RA_non−social_ = 0.54, SD = 0.38; RA_social_ = 0.59, SD = 0.32). To assess the extent to which RA differed between social and non-social contexts, we calculated an index of social risk sensitivity: SRS = RA_social_–RA_non−social_ for each subject. Positive values of SRS (+SRS) signify that the participant exhibits higher risk aversion when a social partner determined the outcome of a risky choice compared to when the outcome was determined by a non-social gamble process. Similarly, negative values of SRS (−SRS) indicate greater risk aversion when the outcome was determined by a non-social versus social process.

To examine risk aversion for social relative to non-social contexts, we compared 16 individuals with +SRS to 14 individuals with −SRS. To confirm that these subgroups indeed differed in risk aversion preference across social and nonsocial conditions, a two-way, repeated measures analysis of variance with GROUP (+SRS, −SRS) and CONDITION (social, non-social) was performed. While no significant effects of GROUP or CONDITION were identified, a significant GROUP × CONDITION interaction [*F*_(1, 28)_ = 17.51, *P* < 0.001] confirmed that risk aversion preferences for social and non-social options differed between the two subgroups. Within the +SRS group, RA_social_ was greater than RA_non−social_ (*Wilcoxon Z* = +3.5; *P* < 0.001) and within the –SRS group, RA_non−social_ was greater than RA_social_ (*Wilcoxon Z* = −3.3; *P* < 0.001).

**Figure 2:**
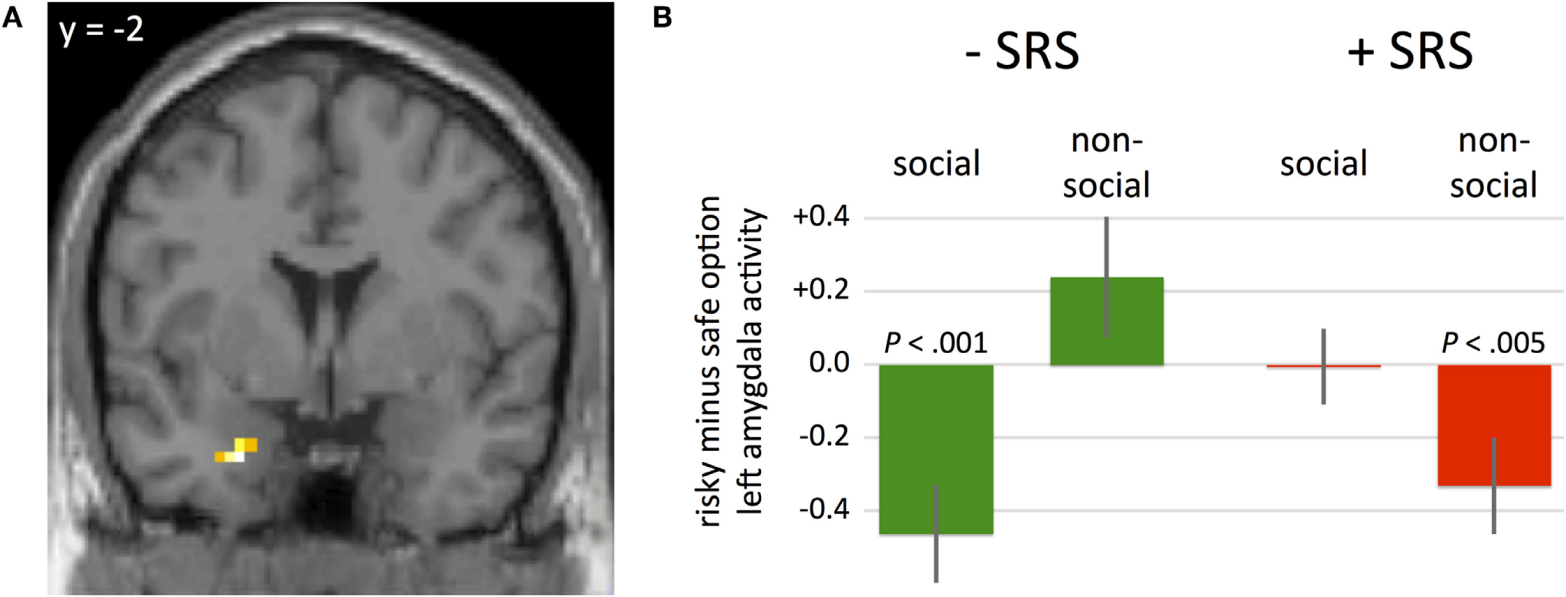
**(A)** A significant effect of the three-way interaction of GROUP (+SRS, −SRS) × CONDITION (social, non-social) × CHOICE (risky option, certain option) was identified in hemodynamic activity in left amygdala (*P*_(FWE, svc)_ < 0.05; peak voxel coordinates: −24, −2, −29). **(B)** Beta weights of GROUP × CONDITION × CHOICE at the peak voxel illustrated in panel **A**. Participants who were more risk averse in the social condition (+SRS) exhibited lower amygdala activity prior to choosing risky relative to certain options in the non-social condition. In contrast, participants who were more risk averse in the non-social condition (−SRS) exhibited lower amygdala activity prior to choosing risky relative to certain options in the social condition.

To identify neural correlates of social risk sensitivity during the decision-making phase of the task, we examined hemodynamic activity within a three-way ANOVA analysis. Specifically, we restricted our analysis to a region-of-interest (ROI) analysis that included the left amygdala as previous reports have implicated this region in both social and risky decision-making processes (Coricelli et al., 2005; Hsu et al., 2005; De Martino et al., 2006; Seymour and Dolan, 2008; Weber and Huettel, 2008). The WFU_Pickatlas (Lancaster et al., 1997) was used to generate an anatomical ROI of the left amygdala (with a dilation factor of 1). Based on this anatomical ROI, eighty-six voxels were included. A significant effect of GROUP (+SRS, −SRS) × CONDITION (social, non-social) × CHOICE (risky option, certain option) on hemodynamic activity was identified in left amygdala (Figure 2A; coordinates: −24, −2, −29; *P*_(FWE, small volume correction)_ < 0.05, z-value = 4.43. Figures 2A and B illustrate how amygdala activity varies by SRS group, condition, and choice. In three of four conditions, amygdala activity is consistent with the SRS bias, despite no overall effects of condition (social vs non-social). For instance, among the subgroup that preferred social over non-social risk (−SRS), greater amygdala activity was observed when choosing the certain versus risky option in the social condition, and the risky versus certain option in the non-social condition. This pattern also holds true for the preference congruent condition among subjects preferring non-social risk: greater amygdala activity was observed in the certain relative to risky option in the non-social condition among subjects in the +SRS group. The only exception to this pattern is for the preference incongruent condition in the +SRS group.

**Figure 3:**
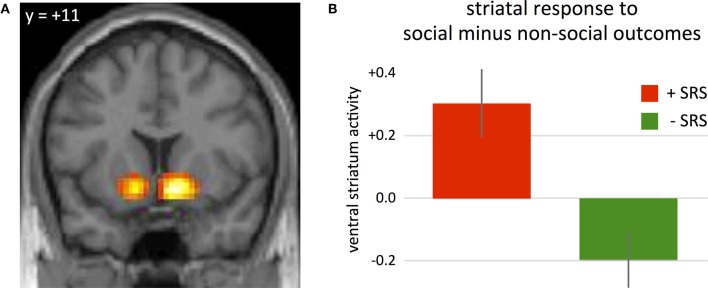
**(A)** Differential sensitivity to reward in social and non-social contexts was observed in the ventral striatum during the presentation of outcome, with higher activity following high-reward outcomes relative to low-reward outcomes (*P*_(FWE, whole brain correction)_ < 0.05, peak voxel coordinates: 9, 11, −11). **(B)** Beta weights of GROUP × OUTCOME interaction at peak voxel within an anatomically-defined region of interest including caudate, putamen, and globus pallidus (*P*_(FWE, svc)_ < 0.05, peak voxel coordinates: 12, 5, −17). A significant two-way interaction revealed that individuals who were more risk averse in non-social contexts (−SRS) exhibited greater striatal activity following non-social outcomes, individuals that were more risk averse in the social contexts (+SRS) showed greater striatal activity following social outcomes.

To examine differential sensitivity to reward in social and non-social contexts, we analyzed the hemodynamic activity at the onset of decision outcomes. We first contrasted high-reward outcomes with low-reward outcomes following risky choices. Consistent with previous studies (Knutson et al., 2001; O'Doherty, 2004; Tobler et al., 2008), hemodynamic activity in the ventral striatum was greater following high-reward outcomes relative to low-reward outcomes across subjects (Figure 3A; 9, 11, −11, *P*_(FWE, whole brain correction)_ < 0.05, z-value = 5.91; −12, 8, −11, *P*_(FWE, whole brain correction)_ < 0.05, z-value = 5.2). An anatomically defined region was used to further examine reward-related activity as a function of group (+SRS, −SRS) and source of outcome (social, non-social). Specifically, the WFU_Pickatlas (Lancaster et al., 1997) was used to generate an anatomical ROI that includes bilateral caudate, putamen, and globus pallidus (with a dilation factor of 2). Based on this anatomical ROI, 4182 voxels were included. A significant two-way interaction revealed that individuals who were more risk averse in social relative to non-social contexts (+SRS) exhibited greater striatal activity following social relative to non-social outcomes, while the −SRS group showed greater striatal activity following non-social relative to social outcomes (Figure 3B; 12, 5, −17, *P*_(FWE, svc)_ < 0.05, z-value = 4.33).

## Discussion

The present study examines behavioral and neuronal differences between evaluating and acting on two sources of risk: one in which outcomes depend on a random non-social process and one in which outcomes depend on the action of another social agent. Values of outcomes and their associated probabilities were known and indistinguishable across social and non-social treatments, allowing us to attribute treatment-related differences to the social or non-social source of risk alone.

Behaviorally, we found that RA in social and non-social contexts were correlated across subjects, which is consistent with the notion that social risk preferences are in part accounted for by risk preferences in non-social contexts. Nevertheless, there is a large majority of unexplained variance that may be accounted for by the distinct risk preferences in social and non-social contexts. In this paper, we focus on this distinction and systematically relate it to neural activity (Figures 2 and 3). From our perspective, the partial concordance and partial discordance of risk parameters observed in our data nicely contributes to the ongoing debate over the shared and unshared variance of risk preference in social versus non-social domains. The correlational result showing that social and non-social RA are related provides support for the notion that trusting behavior is strongly influenced by non-social preferences for risk. This result is consistent with field studies, including Karlan (2005) who found that villagers in Peru who entrust more money in a trust game are also more likely to save less and default more often on loans. Yet other studies (Eckel and Wilson, 2004; Houser et al., 2010) suggest that RA and trust behavior are not strongly related. It could be argued that the similarity between the attitudes toward social and non-social risk is experimentally imposed, as the alteration between non-social and social conditions might prime the subjects to face the social situation as a non-social gamble, or vice versa. However, this suggestion is challenged by two patterns of results.

First, neuroimaging differences between the social and non-social conditions, during both decision and outcomes phases suggest that participants differentiated social and non-social decisions. Consistent with previous studies contrasting social and non-social choice, Table 1 illustrates that greater activity was identified in bilateral fusiform, medial orbitofrontal cortex, bilateral amygdala, and posterior cingulate during the decision phase of the social relative to non-social condition, while greater activity was identified in medial prefrontal cortex during the outcome phase.

**Table 1:** **Social > Non-Social Contrast**.

**Region**	**MNI coordinates**					**Peak**
	***x***	***y***	***z***	**Cluster**	***Z***	**P_FWE_**
**DECISION PHASE**						
R fusiform gyrus	42	−52	−17	1006	7.52	0.001
R parahippocampal gyrus	21	−7	−11	273	5.97	0.001
L fusiform gyrus	−39	−46	−20	683	5.90	0.001
R precuneus	3	−58	31	669	5.85	0.001
L medial frontal gyrus	−9	44	−17	431	5.25	0.006
L superior frontal gyrus	−6	56	28	581	5.12	0.011
R uncus	36	−4	−35	28	4.99	0.022
R middle temporal gyrus	54	−7	−17	400	4.95	0.028
L inferior frontal gyrus	−24	35	−29	11	4.77	0.058
**OUTCOME PHASE**						
L medial frontal gyrus	−6	56	16	38	5.78	0.001
L rectal gyrus	−3	38	−20	19	5.30	0.004
L precuneus	−6	−52	31	17	5.03	0.013
L middle temporal gyrus	−45	−64	22	2	5.01	0.014
R parahippocampal gyrus	18	−7	−14	2	4.91	0.022
R superior temporal gyrus	48	−58	19	6	4.90	0.024
R superior temporal gyrus	54	−58	16	1	4.75	0.045

Second, substantial variability in computed risk aversion between conditions was found across subjects (+SRS and −SRS), and this variability was systematically related to neural activity across subjects during both decisions (Figure 2) and outcomes (Figure 3). Thus, the suggestion that neural computations of risk in social and non-social contexts is isomorphic, is only partially supported by the current data.

A number of behavioral studies have suggested systematic discordance between social and non-socially determined risk. Bohnet et al. (2008) found that participants in six countries had different risk acceptance frequencies for gambles determined by “nature” versus a human partner. Such differences have been primarily attributed to two factors: (i) other-regarding preferences over the allocation of resources (see Rabin, 1993; Fehr and Schmidt, 2002) and (ii) aversion to either betrayal (Bohnet and Zeckhauser, 2004) or exploitation (Fehr et al., 2005). Other-regarding preferences can be quantified by the utility gained or lost by the allocation of resources to others. Thus, the decision-maker might choose to send money to a social partner because of increased utility accrued by the simple act of sharing, regardless of any expectation of repayment. On the other hand, a growing body of research indicates that betrayal aversion, a social counterpart of regret aversion, can deeply influence social behavior (Koehler and Gershoff, 2003; Aimone and Houser, 2012; Bohnet et al., 2008). In this account, betrayal by a social partner confers additional disutility beyond the monetary loss, and the potential for this disutility leads to greater risk aversion in social contexts. While the separable contribution of each of these factors to the social risk sensitivity observed here cannot be assessed within the current design, the joint utility, and disutility leading to either greater or lesser risk aversion in the social condition is evident in limbic activity during the decision phase of the task.

Our neuroimaging findings indicate that differences in RA exhibited by the SRS subgroups depend on differential functional amygdala responses to social and non-social risk. During the decision phase, the amygdala biases behavioral choices in accordance with the underlying social preferences: participants who are socially risk averse show reduced amygdala activity preceding risky non-social choices, but not risky social choices. In contrast, social risk-preferring participants had reduced amygdala activity before risky social choices, but not risky non-social choices. This pattern is reminiscent of the functional role of amygdala in cognitive biases modulated by emotional parameters. For example, De Martino et al. (2006) identified that sensitivity to framing effects (i.e., behavioral changes to isomorphic gambles presented in a positive or negative light) is mediated by amygdala activity. In our experiment, the social and non-social gambles are isomorphic; however, emotional factors such as betrayal aversion and fairness considerations might enter the decision equation thus biasing the behavioral choices. Further, individual differences in social preferences over the allocation of resources has also been shown to scale with activity in the amygdala, suggesting that other-regarding preferences contribute the additional utility or disutility reflected in the behavioral +SRS and –SRS subgroups, respectively (see also Haruno and Frith, 2010).

Hemodynamic responses in the ventral striatum to the outcome of decisions has typically been associated with prediction errors, signaling differences between expected value and observed value at decision outcomes, and the pattern observed here is consistent with such findings (Glimcher, 2011). In addition, both Fleissbach et al. (2007) and Tricomi et al. (2010) demonstrated that striatal responses at the outcome of social decisions are mediated by social comparison considerations. Thus, the increased responsivity of this region during the preference incongruent condition (social in +SRS; non-social in −SRS), suggests that social risk sensitivity is related to increased evaluation of social outcomes. If so, it provides support for the idea that betrayal/exploitation aversion plays an important role in the observed SRS biases. That is, the influence of betrayal/exploitation aversion is most likely to be evident at the outcomes of the decision, when the betrayal (or not) is revealed.

Although these patterns of results indicate that underlying social preferences potentially can influence choice behavior over and above pure risk preferences, there are some potential limitations to consider regarding the interpretation of our findings. Specifically, it might be possible that the differences in RA between conditions (social and non-social) could be attributed to perceptual features of stimuli that differ between conditions, yet are not primarily due to the social versus non-social nature of the two conditions. However, the 3-way interaction observed in the amygdala reflects a further differentiation of risky relative to certain options, which is unlikely to be accounted for by differences between social and nonsocial condition that are unrelated to risk. In addition, the amygdala results were found using ROI analysis and were not whole brain corrected. We used this method as it is generally accepted by the larger scientific community given the small size of this brain structure (De Martino et al., 2006; Haruno and Frith, 2010).

In conclusion, these findings suggest that even in socially minimal situations, investment decisions differ according to the source of uncertainty. This implies not only that decision axioms can be robustly violated when the social element enters the equation but that trust should not be treated us a unitary concept. Social predispositions reflected in amygdala activity during the decision phase as well as differential evaluative mechanisms during the reward outcome phase can lead to diverging behaviors. Future research may establish the underlying factors of individual differences in social responses as well as isolate the effects of pure RA, betrayal aversion, and altruistic considerations on trust behavior.

### Conflict of interest statement

The authors declare that the research was conducted in the absence of any commercial or financial relationships that could be construed as a potential conflict of interest.
